# Factors Influencing the Efficacy of Concurrent Training in Team Sports: A Narrative Review

**DOI:** 10.5114/jhk/216685

**Published:** 2026-02-05

**Authors:** Rafael Tundidor-Duque, Fernando Pareja-Blanco, Milosz Drozd, Lucas A. Pereira, Irineu Loturco

**Affiliations:** 1 Science-Based Training Research Group, Physical Performance and Sports Research Center (CIRFD), Universidad Pablo de Olavide, Seville, Spain.; 2 Faculty of Sports Sciences, Department of Sports and Computer Sciences, Universidad Pablo de Olavide, Seville, Spain.; 3 Centro Universitario San Isidoro, Seville, Spain, Seville, Spain.; 4 Institute of Sport Sciences, The Jerzy Kukuczka Academy of Physical Education in Katowice, Katowice, Poland.; 5 NAR, Nucleus of High Performance in Sport, Sao Paulo, Brazil.; 6 Department of Human Movement Sciences, Federal University of São Paulo, São Paulo, Brazil.; 7 UCAM Research Center for High Performance Sport, UCAM Universidad Católica de Murcia, Murcia, Spain.; 8 FSI, Football Science Institute, Granada, Spain.

**Keywords:** athletic performance, elite athletes, resistance training, muscle strength, sprint speed

## Abstract

Concurrent training (CT)—the integration of strength and endurance exercises within the same session or cycle—is widely implemented in team sports. However, its optimal configuration and the conditions under which the so-called “interference effect” occurs remain subjects of debate. This narrative review critically examines the factors influencing CT efficacy in team sports, emphasizing the roles of training sequence, inter-session recovery, the endurance-training modality, and athletes’ strength levels. Thirteen experimental studies involving male and female athletes from various team sports and competitive levels were analyzed. The evidence suggests that CT may effectively enhance both strength and endurance capacities when properly structured. Performing strength training before endurance training, or separating sessions by at least six hours, appears to minimize neuromuscular fatigue and preserve positive performance adaptations. Conversely, high volumes of endurance training or insufficient recovery periods tend to intensify the interference effects, particularly in speed- and power-related outcomes, which are often more pronounced in top-level athletes. Overall, CT may be a viable strategy for optimizing multiple physical qualities in team-sport environments, provided that its variables are carefully and systematically manipulated. This review also highlights the need for long-term interventions and female-specific studies to refine current practices and strengthen the evidence base in applied high-performance settings.

## Introduction

The physical performance of team-sport players relies on a wide range of abilities, many of which depend on both the aerobic and the anaerobic metabolism (Nader, 2006). Accordingly, team sports typically require the execution of movements that demand high levels of force, such as sprinting and jumping (Suchomel et al., 2016), interspersed with periods of low-intensity activities (i.e., walking and jogging) (Gharbi et al., 2015). Furthermore, strength levels have been shown to be a differentiating factor among athletes of various competitive rankings, with top-level players typically exhibiting greater values than their less skilled counterparts (Soriano et al., 2024). On the other hand, performance in team sports is strongly associated with athletes’ capacity to repeatedly perform high-intensity efforts of a multifaceted nature (e.g., accelerations, decelerations, and multidirectional runs) over the course of a match—physical attributes largely influenced by strength, power, and endurance (Gharbi et al., 2015; Makaruk et al., 2024; Skalski et al., 2024). Therefore, integrating and developing these capacities is essential to achieving success in team sports.

The integration of training strategies aimed at increasing both strength and endurance capabilities is known as concurrent training (CT). Although it is widely implemented in real-world practice (Wang and Bo, 2024), the optimal configuration of both modalities remains unclear, especially for competitive athletes. Interest in this area of research began with the landmark study by Hickson (1980), which reported reduced strength gains in a group of athletes performing CT compared to a group performing only strength training (ST). This outcome was termed the “interference phenomenon”, and since then it has become a major subject of study in sport science (Schumann et al., 2022; Wilson et al., 2012). A recent systematic review by Seipp et al. (2023) concluded that CT was an effective strategy for improving fundamental physical qualities in team-sport players, such as strength, speed, and endurance. Nevertheless, those authors observed that CT-related impairments tended to become more pronounced as athletes’ performance levels increased (Coffey and Hawley, 2017). However, that review also included studies that did not specifically examine the influence of distinct endurance training (ET) strategies (Ramirez-Campillo et al., 2016) as well as studies that compared different ST protocols rather than directly assessing the interference effect of combining ST and ET (Koundourakis et al., 2014). Indeed, those studies did not address the central issues and inconsistencies of CT—namely, whether the combination of both modalities yields superior outcomes compared to unimodal training (i.e., isolated ST or ET) (Loturco et al., 2023), and under what conditions negative interactions between modalities may occur (Petre et al., 2021; Schumann et al., 2022). Moreover, variables such as ET intensity (Monserda-Vilaro et al., 2023), inter-session recovery duration, and the sequencing of ST and ET (Wang and Bo, 2024) may further modulate adaptive responses and thus warrant more detailed investigation. In contrast to previous systematic reviews (Schumann et al., 2022; Wilson et al., 2012) that primarily synthesized evidence under controlled conditions, the present narrative review seeks to place these findings within the real-world context of high-performance sports. In such environments, training strategies are not prescribed solely based on their physical and physiological efficacy, but also on their feasibility and applicability within competitive settings characterized by congested schedules and accumulated training loads.

Therefore, given the persistent uncertainty regarding the optimal manipulation of training variables within CT protocols and considering that recovery-related factors may play a more prominent role than the isolated order of training modalities, the purpose of this narrative review was to examine the differences in training adaptations when comparing CT with unimodal (ST or ET) training strategies in team-sport athletes. Furthermore, this article aimed to address key moderating factors within CT, including session order, the inter-session recovery interval, the ET format, and athletes’ training status, as well as to determine the conditions under which CT may optimize strength- and endurance-related performance outcomes.

## Concurrent Training Interventions

To meet the purpose of this review, we included studies conducted with team-sport players of varying levels and categories (i.e., amateur, semi-professional, or elite) that compared CT protocols either with ET or ST alone, or that evaluated different CT configurations. Only original experimental studies published in peer-reviewed journals and reporting pre- and post-intervention outcomes related to strength, endurance, or speed performance were considered. The literature search was conducted using widely recognized databases (PubMed, Scopus, and Google Scholar), with studies selected based on their theoretical relevance and applied significance, as well as our research group’s experience in this area, as part of a larger research project on the effects of CT in team-sport contexts. In total, thirteen studies were included, involving youth (three studies with under-20 players) and senior athletes (ten studies with adults), both female and male participants, conducted during either the in-season or the off-season period. To address the questions raised above, the present review was organized into the following sections:

1. Studies comparing the effects on strength-related variables of performing ST in isolation versus within a CT program;
2. Research assessing the influence of ET performed alone versus ET combined with ST on endurance-related performance outcomes within a CT approach;
3. Evidence exploring the impact of different CT protocols, such as low-intensity endurance training compared to high-intensity interval training (HIIT), to determine how the nature of the endurance component affects performance outcomes;
4. Studies investigating the effects of manipulating specific variables within CT protocols, such as the order of ST and ET, aiming to identify optimal configurations for maximizing adaptations in both physical qualities; and
5. Analysis of the influence of training status on the adaptations to CT.

### Concurrent Training: Effects on Strength and Speed Abilities

Historically, strength-related variables have been the most affected by the so-called interference effect, notably those linked to strength and speed capacities (Schumann et al., 2022), which are highly relevant to sports performance (Suchomel et al., 2016). Consequently, optimizing CT is of critical importance for the development of these neuromuscular qualities in athletic populations. Although a CT program might be expected to negatively impact maximal strength and sprint speed, to date, some studies have reported no CT-related impairments in these neuromechanical measures (Huiberts et al., 2024; Sabag et al., 2018). In this section, we present a detailed discussion of studies examining the influence of CT approaches on strength and speed performance, as well as the specific contexts in which interference-related effects may be more pronounced. These data are summarized in Table 2. These findings are of particular interest for team sports characterized by congested schedules, in which strength and speed qualities must often be developed and maintained under limited recovery conditions (Petre et al., 2021; Schumann et al., 2022).

**Table 1a:** Summary of studies and associated training protocols examining the effects of concurrent training in team-sport players.

Reference	Subjects	Groups	Details	Duration	Protocols
Arslan et al. (2024)	21 male amateur soccer players	ST + HIIT (7) ST + HIFT (7) C (7)	CT order: ST + ET 15–20’ between sessions	8 weeks 2 x week	ST: squat and BP: 3 x 6 at 70% 1RM/3 x 5 at 85% 1RM, CMJ: 2 x 4/3 x 5, Sprint: 2 x 30 m/3 x 20 m HIIT: 4 x 20”/10” 85% of Yo-Yo distance HIFT: 4 x 20”/10” (burpees, air squats, etc.)
Balabinis et al. (2003)	26 male college basketball players	ET (7) ST (7) ET + ST (7) C (5)	CT order: ET + ST 7 h between sessions	7 weeks 4 x week	ST: HS: 40–90% 1RM, BP: 40–95% 1RM, LP: 40–95% 1RM, PD: 40–95% 1RM ET: Interval training from 85% of HR_max_ to full speed runs
Belkadi et al. (2025)	18 male elite handball players	ST + RSE (8) ST + HIIT (7)	No information	12 weeks	ST (CCT): squat: 3 x 1RM, JS: 3 x 6 at 50% BM, DJ: 3 x 6 RSE: 10 SS 2 x 15 m, 54” rest HIIT: 5 x 30”, 2.5’ rest
Bern et al. (2021)	20 elite female athletes	ST + COMB (10) ST + SEP (10)	CT order: ST + ET COMB: same session SEP: 7 h between sessions	6 weeks 3 ST sessions 2 ET sessions	ST: Multiarticular exercises at 65–90% 1RM ET: Combination of SSGs and linear sprints (6–8’)
Botonis et al. (2016)	14 male elite water polo players	ST + HIIT 4x4 (7) ST + HIIT 16x100 (7)	24 h between sessions	8 weeks ST: 2 x week ET: 2 x week	ST: BP, PD, TP, SP, LP: 4 x 4–5 at 85–90% 1RM HIIT 1: 4 x 4 min at 106% of V4, 3’ active rest HIIT 2: 2 x 8 x 100 m at 106% of V4, 20’’ rest
Enright et al. (2015)	15 male elite soccer players	ST + ET (8) ET + ST (7)	ST + ET: ST (8:45 h) + ET (10:30 h) ET + ST: ET (10:30 h) + ST (14:00 h)	5 weeks 2 x week	ST: HS, DL, SDL, LE: 4 x 6 at 85% 1RM, NHE: 3 x 8 ET: SSG + tec/tac (113’ at 7–10 RPE)
Hennessy and Watson (1994)	56 male rugby and Gaelic soccer players	ST (9) ET (12) ST + ET (10) C (10)	CT: 2 days (ET + ST/ST+ ET)	8 weeks ST: 3 x week ET: 4 x week ST + ET: 5 x week	ST: multiple exercises at 65%–100% 1RM ET: 70% HR_max_ 20–60’, 1 day: Fartlek 15–35’, 1 day: 85% HR_max_, 20–40’

Note: ST = strength training; ET = endurance training; CT = concurrent training; C = control; HIIT = high-intensity interval training; HIFT = high-intensity functional training; COMB = combined training; SEP = separate sessions; BP = bench press; CMJ = countermovement jump; 1RM = one-repetition maximum; HS = half-squat; LP = leg press; PD = pull-down; HR_max_ = maximum heart rate; CCT = complex-contrast training; BM = body mass; DJ = drop jump; RSE = repeated short sprints; SS = shuttle sprints; SSG = small-sided games; TP = triceps press; SP = shoulder press; V4 = velocity associated with 4 mmol•L^-1^ of blood lactate; DL = deadlift; SDL = straight-leg deadlift; LE = leg extension; NHE = Nordic hamstring exercise; RPE = rate of perceived exertion

**Table 1b:** Summary of studies and associated training protocols examining the effects of concurrent training in team-sport players.

Reference	Subjects	Groups	Details	Duration	Protocols
McGawley and Anderson (2013)	18 male semi and fully professional soccer players	HIIT + ST (9) ST + HIIT (9)	5’ between sessions	5 weeks 3 x week	HIIT: HIIT or SSGs with or w/o the ball 90–95% HR_max_ ST: 5–6 exercises: 2–3 x 5–10 at 75–90% 1RM
Petre et al. (2018)	16 male high levels ice-hockey and rugby players	ST + CET (8) ST + HIIT (8)	CT order: ST + ET 15’ between sessions ET: cycling	6 weeks 3 x week	ST: squat: 2–5 sets at 80–90% 1RM CET: 40–80’ at 70% VO_2max_ HIIT: 1–3 x 8 x 20’’ at 150% VO_2max_
Petre et al. (2023)	23 elite male bandy players	ST + HIIT (8) HIIT + ST (7) ST (7)	10’ between sessions ET: cycling	7 weeks 2 x week	ST: squat kBox: 4 x 6 HIIT: 2–4 x 8 x 20”/10” at 130% MAP
Robineau et al. (2016)	58 amateur male rugby players	ST (10) CT0h (15) CT6h (11) CT24h (12) C (10)	CT order: ST + ET CT0-h: same session CT6-h: 6 h between sessions CT24-h: 24 h between sessions	7 weeks 2 x week	ST: BP, BR, HS, LP: 3–4 x 3–10 RM ET: 6 x 15”/15” at 120% MAS
Robineau et al. (2017)	35 amateur male rugby 7 players	ST (11) ST + HIIT (9) ST + SIT (10)	CT order: ST + ET 24 h between sessions	8 weeks 2 x week	ST: HS, DL, LE, BP, BR: 3 x 3–10 at 70–90% 1RM HIIT: 2 x 8–12 min of 30/30’’ at 100% MAS SIT: 4–8 x 30” all out, 4’ rest
Sanchez-Sanchez et al. (2019)	24 male regional level athletes (soccer and basketball)	CT (12) HIIT (12)	CT order: ST + HIIT 10’ between sessions	5 weeks 2 x week	ST: BL and HK iso-inertial conical pulley: 2–3 x 6 HIIT: 2 x 8 x 30”/30” at 90–100% HR_max_ 3’ rest

Note: ST = strength training; ET = endurance training; CT = concurrent training; C = control; HIIT = high-intensity interval training; SIT = sprint interval training; BP = bench press; 1RM = one-repetition maximum; HS = half-squat; LP = leg press; HR_max_ = maximum heart rate; SSG = small-sided games; DL = deadlift; LE = leg extension; VO_2max_ = maximal oxygen uptake; CET = continuous endurance training; MAP = maximal aerobic power; MAS = maximal aerobic speed; BR = bench row; BL = back lunge; HK = hamstring kick

**Table 2:** Summary of the effects of different concurrent training configurations on neuromuscular capacities in team-sport players.

Reference	Subjects	Groups	Duration	Neuromuscular adaptations
Arslan et al. (2024)	21 male amateur soccer players	ST+ HIIT (7) ST + HIFT (7) C (7)	8 weeks 2 x week	HIIT: ↑1RM squat, BP, ↑CMJ, ↑20-m sprint HIFT: ↑1RM squat, BP, ↑CMJ C: No significant changes
Balabinis et al. (2003)	26 male college basketball players	ET (7) ST (7) ET + ST (7) C (5)	7 weeks 4 x week	ST and CT: ↑1RM HS, BP, LP, PD, ↑CMJ ET: No significant changes C: ↓1RM LP
Belkadi et al. (2025)	18 male elite handball players	ST + RSE (8) ST + HIIT (7)	12 weeks	RSE: ↑1RM squat, ↑RFD, ↓5-m sprint, ↑20–30-m sprint, ↓RSA best HIIT: ↑1RM squat, ↑RFD, ↑5JT, ↑20–30-m sprint
Bern et al. (2021)	20 elite female athletes	ST + COMB (10) ST + SEP (10)	6 weeks 3 ST sessions 2 ET sessions	Both groups: ↑1RM squat, BP, ↑10-m sprint
Botonis et al. (2016)	14 male elite water polo players	ST + HIIT 4x4 (7) ST + HIIT 16x100 (7)	8 weeks ST: 2 x week ET: 2 x week	Both groups: ↑1RM BP
Enright et al. (2015)	15 male elite soccer players	ST + ET (8) ET + ST (7)	5 weeks 2 x week	ST + ET: ↑1RM HS, ↑IMVC, ↑SJ ET + ST: ↑1RM HS, ↑IMVC, ↑SJ, ↑10-m sprint
Hennessy and Watson (1994)	56 male rugby and Gaelic soccer players	ST (9) ET (12) ST + ET (10) C (10)	8 weeks ST: 3 x week ET: 4 x week ST + ET: 5 x week	ST: ↑1RM BP, squat (higher increase), ↑VJ, ↑20-m sprint CT: ↑1RM BP, squat ET and C: No significant changes
McGawley and Anderson (2013)	18 male semi and fully professional soccer players	HIIT + ST (9) ST + HIIT (9)	5 weeks 3 x week	Both groups: ↑1RM squat, ↑CMJ, ↑10-m sprint
Petre et al. (2018)	16 male high levels ice-hockey and rugby players	ST + CET (8) ST + HIIT (8)	6 weeks 3 x week	CET and HIIT: ↑1RM squat
Petre et al. (2023)	16 male high-level ice hockey and rugby players	ST + HIIT (8) HIIT + ST (7) ST (7)	7 weeks 2 x week	ST + HIIT: ↑MIF squat HIIT + ST: ↑MIF squat, ↓CMJ ST: ↑MIF squat
Robineau et al. (2016)	58 amateur male rugby players	ST (10) CT0h (15) CT6h (11) CT24h (12) C (10)	7 weeks 2 x week	ST, CT0, CT6, CT24: ↑1RM BP, BR, HS, ↑CMJ C: No significant changes
Robineau et al. (2017)	35 amateur male rugby sevens players	ST (11) ST + HIIT (9) ST + SIT (10)	8 weeks 2 x week	ST, HIIT, SIT: ↑1RM BP, BR, squat, ↑CMJ
Sanchez-Sanchez et al. (2019)	24 male regional level athletes (soccer and basketball)	CT (12) HIIT (12)	5 weeks 2 x week	HIIT: No significant changes CT: ↑COD, ↑CMJ

Note: ST = strength training; ET = endurance training; CT = concurrent training; C = control; HIIT = high-intensity interval training; HIFT = high-intensity functional training; SIT = sprint interval training; RSE = repeated short sprints; COMB = combined training; SEP = separate sessions; CET = continuous endurance training; BP = bench press; CMJ = countermovement jump; 5JT = five-jump test; 1RM = one-repetition maximum; HS = half-squat; PD = pull-down; RFD = rate of force development; LP = leg press; BR = bench row; MAS = maximal aerobic speed; MIF = maximal isometric force; IMVC = isometric maximal voluntary contraction; SJ = squat jump; VJ = vertical jump; RSA = repeated-sprint ability; COD = change-of-direction speed. ↑ = significant improvement; ↓ = significant decrease

Different ST methods and CT configurations have been used. For example, Balabinis et al. (2003) compared a CT group performing ET in the morning and ST in the afternoon (7 hours apart) with an ST-only group of amateur basketball players. The ST protocol included four exercises (i.e., bench press [BP], half-squat [HS], leg press [LP], and pull-down [PD]) at intensities ranging from 40% to 95% one repetition maximum (1RM), while ET consisted of interval training from the 85% maximum heart rate (HR_max_) to all-out sprints. Both groups improved the strength-power-related variables (1RM in the HS, BP, LP and PD exercises and countermovement jump [CMJ] height) similarly. Robineau et al. (2016) investigated three CT protocols differing in the rest interval between ST and ET (0, 6, and 24 h), along with an ST-only group in amateur rugby players. ET consisted of running intervals at 120% maximal aerobic speed (MAS), and ST involved four exercises at 70–90% 1RM, performed before ET. Those authors reported that when sessions were separated by 6 hours, no differences emerged between CT and ST-only groups in the 1RM BP, the bench row, the squat, or CMJ height. In a subsequent study, two CT protocols—one with ET performed as sprint interval training (SIT) (30-s sprints with 4 min of rest) and the other using short intervals (30-s intervals at 100% MAS with 30 s of recovery)—both with a 24-h separation from ST (four exercises at 70–90% 1RM) were compared with an ST-only control group of amateur rugby players (Robineau et al., 2017). No significant differences in strength-related variables were observed, except for concentric torque at low speeds (60°•s^−1^ at isokinetic knee extension), where the SIT group showed smaller changes compared with the other groups. In summary, current evidence suggests that the interference phenomenon can be mitigated through careful manipulation of training variables. When ST and ET are executed within the same training session, it is recommended that ST precedes ET to minimize the detrimental effects of accumulated fatigue on neuromuscular performance. Conversely, when it is possible to separate the two modalities by at least six hours, session sequencing appears to have little to no impact on performance outcomes. Thus, the available evidence highlights the importance of strategic session planning to optimize adaptations in CT contexts. In real-world team-sport settings, such systematic planning must also account for accumulated training and match loads, which may further influence acute and chronic fatigue and recovery dynamics (Robineau et al., 2016).

On the other hand, Hennessy and Watson (1994) examined the effects of a CT protocol versus an ST protocol in amateur rugby and Gaelic football players. The ST program comprised three sessions per week, each including distinct lower- and upper-body exercises (e.g., arm curl, back squat, BP, lunge, and PD), whereas ET consisted of two continuous and two interval training sessions at 70–85% HR_max_. The CT group (who trained five times per week) exhibited smaller improvements in lower-body strength and jump height compared to the ST group, although upper-body strength gains were similar between the two groups. In addition, 20-m sprint performance improved only in the ST group. Similarly, in the previously mentioned study by Robineau et al. (2016), the CT group that performed both training modalities consecutively without rest (i.e., CT0) demonstrated smaller improvements in HS, BP, and bench row strength than the other CT groups and the ST group. Petre et al. (2023) compared the effects of two distinct CT configurations—one with ET (i.e., HIIT) performed prior to ST and the other in the reverse order (with a 10-min rest interval between modalities)—with a group performing only ST in elite bandy players (i.e., an ice-based team sport played with skates, sticks, and a ball, on a large field). The ST protocol consisted of four sets of squats using a flywheel device, whereas the HIIT protocol involved short cycling intervals at 130% MAS. All groups improved maximum isometric force in the squat exercise equally; however, the group that performed HIIT prior to ST showed a significant reduction in CMJ performance. Additionally, the HIIT + ST group exhibited lower power output during ST sessions, suggesting that prior HIIT may have induced a transient neuromuscular fatigue state, likely exacerbated by residual metabolic stress. Belkadi et al. (2025) compared the effects of two CT structures in elite handball players: one including short shuttle sprints and the other comprising 30-s sprints with directional changes. In both interventions, ST consisted of complex-contrast training, which consisted of four exercises of varying intensities, ranging from heavy-load exercises (up to 85% 1RM) to body mass movements. Although both groups improved strength-related variables (e.g., squat-1RM and the rate of force development), the short-sprint group showed a significant decrease in 5-m sprint performance and in the peak sprint speed achieved during a repeated sprint ability (RSA) test. Thus, when both modalities are executed within the same training session (with less than one hour of rest) or under conditions of high ET volumes (e.g., five training days per week including more than two high-intensity sessions), the likelihood of delayed or compromised adaptations appears to increase, especially for ST-related variables such as 1RM, vertical jump height, and sprinting speed. It is important to emphasize that these effects may also be influenced by the excessive demands imposed during the initial training block (i.e., first training session) and by insufficient recovery time-frames, rather than being solely attributed to a conceptual interference phenomenon (Petre et al., 2023). This distinction is critical in elite team sports, where high training and match density can also intensify recovery-related constraints (Seipp et al. (2023)).

### Concurrent Training: Effects on Endurance Performance

Although substantial evidence indicates that ST can improve endurance-related variables, such as running economy and anaerobic capacity (Rønnestad and Mujika, 2014), most of these findings pertain to individual sports, where there is minimal interference from additional factors such as technical-tactical training components (e.g., small-sided games [SSGs]) (Casamichana et al., 2014; Karahan, 2020). In addition, distinct ET variables (e.g., volume, intensity, and training strategy) play an important role in CT adaptations (Sabag et al., 2018). Accordingly, this section details the influence of CT on endurance performance outcomes (additional information is presented in Table 3). Of note, in team-sport environments, endurance-oriented stimuli are frequently imposed through both training sessions and matches, thus reinforcing the importance of integrating CT within real-world performance demands. For example, Balabinis et al. (2003) showed in amateur basketball players that the CT group achieved nearly double the improvement in maximal oxygen uptake (VO_2max_) compared with the ET group. The CT group performed ET in the morning and, after a 7-h recovery period, completed the ST session, a scheduling strategy that effectively minimized negative interactions between the two modalities. Likewise, Hennessy and Watson (1994) found no meaningful differences between CT and ET in amateur rugby players, possibly due to the greater training volume accumulated by the CT group compared with the unimodal groups. In line with these findings, Sanchez-Sanchez et al. (2019) demonstrated that combining ET with prior ST—using multiple lower-body exercises on an inertial device—did not compromise training outcomes compared with ET performed alone via HIIT in regional-level basketball and soccer players. The CT group showed greater improvements in change of direction performance and the RSA, whereas the ET group achieved superior gains in a 20-m sprint test. Overall, when comparing endurance adaptations between CT and ET, well-designed ST appears capable of further enhancing ET-induced adaptations.

**Table 3:** Summary of the effects of different concurrent training configurations on endurance-related performance markers in team-sport players.

Reference	Subjects	Groups	Duration	Endurance adaptations
Arslan et al. (2024)	21 male amateur soccer players	ST+ HIIT (7) ST + HIFT (7) C (7)	8 weeks 2 x week	HIIT and HIFT: ↑VO_2max_
Balabinis et al. (2003)	26 male college basketball players	ET (7) ST (7) ET + ST (7) C (5)	7 weeks 4 x week	ST: ↑Wingate ET: ↑VO_2max_ CT: ↑Wingate, ↑VO_2max_ Control: No significant changes
Belkadi et al. (2025)	18 male elite handball players	ST + RSE (8) ST + HIIT (7)	12 weeks	RSE: No significant changes HIIT: ↑TTE, ↑VO_2max_
Bern et al. (2021)	20 elite female athletes	ST + COMB (10) ST + SEP (10)	6 weeks 3 ST sessions 2 ET sessions	Both groups: ↑1200-m speed
Botonis et al. (2016)	14 male elite water polo players	ST + HIIT 4x4 (7) ST + HIIT 16x100 (7)	8 weeks ST: 2 x week ET: 2 x week	HIIT 4x4: ↑V4 speed, ↑V5 speed, ↑V10 speed HIIT 16x100: ↑V10 speed, ↓V5 speed
Hennessy and Watson (1994)	56 male rugby and Gaelic soccer players	ST (9) ET (12) ST + ET (10) C (10)	8 weeks ST: 3 x week ET: 4 x week ST + ET: 5 x week	ET, CT: ↑VO_2max_ ST: No significant changes Control: ↓VO_2max_
McGawley and Anderson (2013)	18 male semi and fully professional soccer players	HIIT + ST (9) ST + HIIT (9)	5 weeks 3 x week	Both groups: ↑Yo-Yo distance
Petre et al. (2018)	16 male high-level ice hockey and rugby players	ST + CET (8) ST + HIIT (8)	6 weeks 3 x week	CET: No significant changes HIIT: ↑ VO_2max_
Petre et al. (2023)	23 elite male bandy players	ST + HIIT (8) HIIT + ST (7) ST (7)	7 weeks 2 x week	ST + HIIT and HIIT + ST: ↑ VO_2max_ ST: No significant changes
Robineau et al. (2016)	58 amateur male rugby players	ST (10) CT0h (15) CT6h (11) CT24h (12) C (10)	7 weeks 2 x week	ST: No significant changes CT0, CT6: ↑VO_2max_ CT24: ↑VO_2max_ (higher increase) C: ↓VO_2max_
Robineau et al. (2017)	35 amateur male rugby sevens players	ST (11) ST + HIIT (9) ST + SIT (10)	8 weeks 2 x week	ST: No significant changes HIIT: ↑VO_2max,_ ↑MAS SIT: ↑VO_2max_, ↑MAS (higher increase), ↓RSA
Sanchez-Sanchez et al. (2019)	24 male regional level athletes (soccer and basketball)	CT (12) HIIT (12)	5 weeks 2 x week	HIIT: ↓20-m shuttle run test CT: ↑RSA

Note: ST = strength training; ET = endurance training; CT = concurrent training; C = control; HIIT = high-intensity interval training; HIFT = high-intensity functional training; SIT = sprint interval training; COMB = combined training; SEP = separate sessions; RSE = repeated short sprints; V4 = velocity associated with 4 mmol of blood lactate; V5 = velocity associated with 5 mmol of blood lactate; V10 = velocity associated with 10 mmol of blood lactate; V10–5 = difference in velocity between blood lactate concentrations of 5 and 10 mmol; CET = continuous endurance training; MAP = maximal aerobic power; MAS = maximal aerobic speed; TTE = time to exhaustion; VO_2max_ = maximal oxygen uptake; RSA = repeated-sprint ability. ↑ = significant improvement; ↓ = significant decrease

Belkadi et al. (2025) reported that the long-sprint group achieved greater gains in total time during an RSA test and improved time to exhaustion, indicating that the configuration of ET can induce specific adaptations within the context of CT. In the study by Robineau et al. (2017) in amateur rugby players, two CT protocols were evaluated: one involving short-interval ET at 100% MAS and the other combining SIT (30-s sprints) with ST completed before ET, using four exercises at 70–90% 1RM. The SIT approach proved to be more effective in enhancing peak VO_2max_ and RSA performance, but appeared to interfere more with strength development, particularly at low movement velocities (60°•s^−1^ at isokinetic knee extension). Petre et al. (2018) investigated two distinct CT protocols in highly trained ice hockey players. The same ST was performed with 2–5 sets of heavy squats (above 80% 1RM). One group performed ET in the form of HIIT (20-s intervals at 150% MAS), while the other undertook continuous training at 70% VO_2max_, both on a cycle ergometer. Improvements were similar across all variables except for VO_2max_, which increased exclusively in the HIIT group. Arslan et al. (2025) examined amateur soccer players’ responses to two different CT protocols. Both groups completed the same ST protocol, incorporating heavy-load squat and BP exercises, complemented by CMJs and sprints. One group performed ET in the form of HIIT, consisting of 20-s running intervals covering 85% of the Yo-Yo test final distance, whereas the other group followed the same 20-s/10-s work-rest structure using calisthenic exercises (e.g., burpees and jumping jacks). Both groups exhibited comparable enhancements in VO_2max_; however, only the HIIT group improved 20-m sprint performance. Similarly, Botonis et al. (2016) compared the effects of two HIIT-based CT protocols in elite water polo players, both using identical ST protocols, which included BP, LP, PD, shoulder press, and triceps press exercises at 85–90% 1RM. Following the first protocol (HIIT4), athletes swam 4-min bouts at 106% of the velocity corresponding to a 4 mmol•L^-1^ blood lactate concentration (V_4_), while in the second protocol (HIIT100), they performed 100-m efforts at the same intensity. The HIIT4 protocol elicited greater improvements in swimming velocities associated with various lactate thresholds. In general, the configuration of ET appears to directly and specifically influence the adaptations derived from CT. Therefore, it is recommended that the specific demands of the sport be carefully considered when selecting and structuring ET and ST variables within CT programs (Dolan et al., 2024).

## Influence of Temporal Variables in Concurrent Training

Several reviews highlight the substantial impact of temporal variables—specifically, exercise order and inter-session recovery—on CT adaptations (Murlasits et al., 2018; Schumann et al., 2022; Wang and Bo, 2024). In team sports, where multiple physical qualities are trained simultaneously and competitive schedules are highly congested, optimizing these variables is essential. Accordingly, these temporal considerations should be interpreted within a comprehensive framework of load accumulation and recovery management typical of high-performance sport settings (Petre et al., 2021; Schumann et al., 2022).

### Influence of Recovery Interval Duration between Sessions

Robineau et al. (2016) examined the effects of varying rest intervals between ST and ET (0, 6, and 24 h). Findings indicated that a 6-h recovery period was sufficient for strength-related outcomes to improve similarly to a 24-h interval; however, the greatest improvement in VO_2max_ occurred in the 24-h separation group. In a related study, Bern et al. (2021) compared elite female rugby players performing CT within the same training session versus those performing ST first (i.e., six exercises at 65–90% 1RM), followed by ET (i.e., SSGs combined with sprint intervals) seven hours later. Both groups improved equally in strength- and endurance-related variables. Current evidence suggests that separating ST and ET into different days or by at least six hours may optimize strength adaptations, whereas a 24-h interval appears more favorable for enhancing VO_2max_. From an applied perspective, this is unsurprising, as positive adaptations in physical performance are generally enhanced when distinct training stimuli (e.g., ST and ET) are strategically separated, with longer intervals between sessions (i.e., ≥ 6 h and up to ~24 h) likely providing additional benefits (Robineau et al., 2016). In applied practice, complementary monitoring of external and internal loads (e.g., GPS-derived metrics, session-RPE, or heart rate-based measures) may help practitioners contextualize these recovery-related effects without altering the fundamental structure of CT programs (Casamichana et al., 2014; Seipp et al. (2023)). Nonetheless, given the limited number of available studies and the practical challenges of prescribing ST and ET sessions on separate days or with extended recovery intervals, as well as implementing adequate recovery strategies in team-sport environments (Coffey and Hawley, 2017; Petre et al., 2021; Schumann et al., 2022), definitive conclusions cannot yet be drawn.

### Influence of Training Status

Another moderating factor discussed in the literature is athletes’ training status (Coffey and Hawley, 2017; Huiberts et al., 2024), particularly strength levels typically assessed via 1RM testing (Santos-Junior et al., 2021). While Coffey and Hawley (2017) hypothesized that the CT-related impairments might be more pronounced in individuals with greater training experience, other investigations have not supported this assumption, provided that sufficient recovery time is allowed between sessions (Schumann et al., 2022). Hence, the following section presents results categorized according to strength levels.

Highly trained athletes are defined as those with a 1RM in the squat exceeding 150% of body mass or more than 120% in the BP for men (Santos-Junior et al., 2021). Studies comparing the effects of ST alone versus CT (Robineau et al., 2016; 2017) found no negative influences on strength adaptations when at least six hours of rest were provided between sessions. Similarly, research examining the influence of the ET format (Botonis et al., 2016; Petre et al., 2018; Robineau et al., 2017) reported no detrimental effects on strength adaptations, regardless of whether both modalities were completed in the same training session (Petre et al., 2018) or on alternate days (Botonis et al., 2016; Robineau et al., 2017). Enright et al. (2015) noticed that the ET + ST group achieved superior results, although with a longer rest interval (120 min vs. 30–45 min) between sessions. Overall, these findings suggest that highly trained athletes can optimize CT adaptations by manipulating variables such as inter-session recovery duration.

Advanced athletes are classified as those with a 1RM in the squat between 120% and 150% of body mass in men and between 100% and 130% in women (Santos-Junior et al., 2021). Studies comparing different ET formats (Arslan et al., 2025; Belkadi et al., 2025) observed training-specific improvements: groups performing running or sprint drills with shorter rest intervals exhibited greater sprint-related gains, while those using longer rest intervals showed greater VO_2max_ enhancements. Hennessy and Watson (1994) reported reduced strength gains in the CT group compared with the ST group but similar endurance improvements (i.e., greater VO_2max_ values) between CT and ET. Mcgawley and Andersson (2013) found no significant differences when altering the order of the training modalities, and Bern et al. (2021) reported equivalent adaptations whether CT was performed within the same training session or separated by a 7-h interval.

Intermediate athletes are defined as those with a 1RM in the squat exercise ranging from 80% to 120% of body mass (Santos-Junior et al., 2021). Balabinis et al. (2003) reported that the CT group, performing ET followed by ST with a 7-h interval between sessions achieved the most balanced improvements across all assessed variables compared with ST and ET executed in isolation. These findings suggest that training status, as assessed via relative strength levels, does not play a major role in influencing adaptations to CT. Instead, the modulation of training outcomes (and, consequently, physical and technical performance) seems to depend primarily on the specific configuration of training variables, such as the rest interval between sessions, which should therefore be carefully considered in both research and applied practice.

### Limitations

Considering the characteristics of the specific training sessions in team sports, all studies that analyzed the effects of strength or speed training sessions are, in essence, CT-based programs. Nevertheless, since the aerobic-based ET content is not always controlled or manipulated in terms of volume, intensity, and intermittent phases (Coffey and Hawley, 2017; Dolan et al., 2024; Wilson et al., 2012), it is not possible to precisely determine the superiority of one training program over the other. In addition, the available literature is characterized by a limited number of longitudinal interventions lasting longer than 12 weeks, which restricts inferences about the long-term (i.e., chronic) responses of different CT configurations. Moreover, the control of concurrent technical and tactical training sessions (e.g., small-sided games), which represent a substantial component of the global training load in team sports, is often insufficiently reported or standardized. For this reason, certain specific studies were not considered in this narrative review, which consequently limits the findings reported here.

## Practical Implications and Future Research Directions

In the context of team sports, which are characterized by highly congested schedules and repeated high-intensity activities (Gharbi et al., 2015; Seipp et al., 2023), CT may represent a practical and effective approach, as it allows the simultaneous development of multiple physical capacities and may be preferable to omitting training of a given capacity (e.g., strength- or endurance-related qualities) due to time constraints. However, this is strongly dependent on the athlete’s performance level, with top-level athletes being much more susceptible to the negative (or absent) effects of CT than their less specialized peers, especially regarding speed- and power-related abilities (Blechschmied et al., 2024; Huiberts et al., 2024; Wang and Bo, 2024). In light of these findings, several practical recommendations should be considered to maximize the benefits of CT and minimize potential interference-related outcomes:

1. When possible, ST should be performed first, as performing ET first may impair the quality of the strength session. If this is not feasible, the longest possible rest interval between sessions should be allowed.
2. When ST is conducted first, a 6-h rest period appears sufficient to preserve strength and speed gains.
3. ET should be selected to match the specific demands of the sport, as adaptations will be specific to the intensity and modality employed.
4. Currently, there is no clear evidence to suggest that training status constitutes a key modulatory factor in CT.

Future research should focus on female athletes, as only one study involving women was included in this review (Bern et al., 2021), and on long-term interventions, given that the mean study duration in the current evidence base is approximately 7 weeks. Longer studies would better replicate real-world sport settings and competitive demands.

## Conclusions

This narrative review examined the effects of CT in comparison with unimodal ST or ET in team-sport athletes, with particular attention given to the impact of temporal variables, ET configuration, and training status. Overall, the available evidence indicates that CT can effectively improve or maintain both strength- and endurance-related qualities when key training variables are appropriately managed. In this context, strong evidence supports prioritizing ST before ET when both sessions are performed on the same day under conditions of high training density or limited recovery. Moreover, separating ST and ET sessions by at least six hours appears sufficient to preserve strength and speed adaptations, whereas longer intervals (≥ 6 h up to 24 h) may offer additional benefits when the primary goal is to maximize aerobic adaptations, such as improvements in VO_2max_. Importantly, ET configuration plays a central role in shaping CT outcomes, as adaptations are highly specific to the modality, intensity, and structure of ET. This reinforces the need to tailor ET selection to the physiological demands of each sport. Contrary to traditional assumptions, training status alone does not seem to be a decisive factor driving CT-related impairments in neuromechanical measures, provided that recovery time and total training load are adequately controlled. Instead, the combined influence of session order, the adequate recovery interval, and accumulated training demands appears to be the primary determinant of adaptive responses. Taken together, these findings suggest that the interference phenomenon should not be viewed as an inevitable consequence of CT, but rather as a context-dependent outcome largely affected by programming decisions and recovery management. By synthesizing current evidence into clear and applicable recommendations, this review offers a practical framework to support practitioners in optimizing the CT prescription within the complex and congested environments typical of contemporary sports.
